# When Phase Contrast Fails: ChainTracer and NucTracer, Two ImageJ Methods for Semi-Automated Single Cell Analysis Using Membrane or DNA Staining

**DOI:** 10.1371/journal.pone.0151267

**Published:** 2016-03-23

**Authors:** Simon Syvertsson, Norbert O. E. Vischer, Yongqiang Gao, Leendert W. Hamoen

**Affiliations:** 1 Centre for Bacterial Cell Biology, Newcastle University, Newcastle upon Tyne, Richardson Road, Newcastle, NE2 4AX, United Kingdom; 2 Swammerdam Institute for Life Sciences, University of Amsterdam, Science Park 904, 1098 XH Amsterdam, The Netherlands; Loyola University Chicago, UNITED STATES

## Abstract

Within bacterial populations, genetically identical cells often behave differently. Single-cell measurement methods are required to observe this heterogeneity. Flow cytometry and fluorescence light microscopy are the primary methods to do this. However, flow cytometry requires reasonably strong fluorescence signals and is impractical when bacteria grow in cell chains. Therefore fluorescence light microscopy is often used to measure population heterogeneity in bacteria. Automatic microscopy image analysis programs typically use phase contrast images to identify cells. However, many bacteria divide by forming a cross-wall that is not detectable by phase contrast. We have developed ‘ChainTracer’, a method based on the ImageJ plugin ObjectJ. It can automatically identify individual cells stained by fluorescent membrane dyes, and measure fluorescence intensity, chain length, cell length, and cell diameter. As a complementary analysis method we developed 'NucTracer', which uses DAPI stained nucleoids as a proxy for single cells. The latter method is especially useful when dealing with crowded images. The methods were tested with *Bacillus subtilis* and *Lactococcus lactis* cells expressing a GFP-reporter. In conclusion, ChainTracer and NucTracer are useful single cell measurement methods when bacterial cells are difficult to distinguish with phase contrast.

## Introduction

Isogenic populations of bacteria show a remarkable variability in behavior, especially in challenging growth conditions. For example, some cells can become motile, whereas others might become genetically competent or form spores [1]. In biofilms, differentiation into various cell types has also been well-documented [2]. This cellular variation within isogenic populations is common and often a bet-hedging strategy, as it prepares the species for unforeseen environmental changes [3]. It is therefore important to study bacterial gene expression at a single cell level. Flow cytometry has been used for this [4,5], but due to the small size of bacteria, this technique requires a relatively strong fluorescence reporter. Flow cytometry is also impractical when dealing with bacteria that form cell-chains. Therefore, many single-cell gene regulation studies with bacteria use fluorescence light microscopy. To obtain data from sufficient numbers of cells, it is desirable to have automatic analysis software that can interpret the microscopy images. Several software packages have been developed to do this, including *CellProfiler*, *MicrobeTracker* and plugins for ImageJ like *TLM-Quant* [6–8]. These methods use thresholding of the phase contrast image to outline cells. This works well with bacteria such as *Escherichia coli*, *Salmonella typhimurium* or *Caulobacter crescentus*, which show a visible constriction when dividing. However, there are other bacteria for which cell division is difficult to follow using phase contrast. For example *Bacillus subtilis* divides by forming a cross-wall (septum) that cannot be observed by phase contrast. It is therefore difficult to define the boundaries of discrete cells with phase contrast in this species. Moreover, during exponential growth, *B*. *subtilis* cells do not immediately separate after septum synthesis has completed and they often form long cell-chains. This also hinders the use of phase contrast images to identify cells

Here, we describe two methods that enable semi-automated single cell measurements of bacterial cells that do not show clear cell division in phase contrast images. The first method is called 'ChainTracer' and uses membrane stain images to define cell boundaries within a chain. For cases where ChainTracer cannot be used because images are too crowded, a second method called 'NucTracer' has been developed that uses fluorescently stained nucleoids as a proxy for single cells. Both methods run under the plugin ‘ObjectJ’, which in turn is connected to the popular Java-based image processing program ImageJ [9]. ObjectJ supports non-destructive hierarchical marking, and integrates analyses across many multi-channel images, while maintaining active links between marked images. This enables easy navigation between results and raw data.

The use of ChainTracer and NucTracer is contextualized in this paper by measuring motility development in a *B*. *subtilis* culture. *B*. *subtilis* exhibits a wide range of adaptations used to survive in soil, its natural habitat. Most of these differentiation processes are only activated in a subset of cells within the population. Examples of this bimodal regulation are motility, natural competence, and sporulation [3,10–12]. Motility is switched on by induction of *sigD*, coding for the sigma factor responsible for transcription of the motility genes [13]. SigD activates its own expression and this positive feedback leads to a bimodal induction of motility during the exponential growth phase [14]. The induction of motility is accompanied by the production of cell wall autolysins that act to release motile cells from cell-chains [15]. The heterogenic induction of motility presents a convenient case to test single cell analyses with the two ObjectJ methods. In addition, we show how ChainTracer can be used to measure cell-chain length. Finally, cellular heterogeneity of *Lactococcus lactis* was measured using NucTracer. This bacterium grows as cocci and it shows that Nuctracer can be used with morphologically different bacteria.

## Materials and Methods

### Strains and media

Experiments were carried out using *B*. *subtilis* wild type strain 168CA [16] containing the P_*hag*_*-gfp* (*cat)* reporter fusion in the *amyE* locus obtained from strain DS901 [10] (resulting strain BSS339). The strain was constructed using standard protocols for inducing natural competence [17], using the laboratory strain 168CA as recipient. Samples were grown overnight in casein hydrolysate (CH) medium [18], supplemented with 1% glucose to inhibit sporulation, then diluted 20 times into fresh CH medium. After growth to OD_600_ ~0.5, samples were diluted to OD_600_ ~0.05 in fresh pre-warmed CH medium to start the culture. *L*. *lactis* MG1363 and *L*. *lactis* M1, which contains the P_*cel*_-GFP reporter fusion [19], were grown overnight in LB medium, diluted 20 times into fresh LB medium, and grown until OD_600_ ~0.6.

### Microscopy

Microscope slides were made using 1% agarose in deionized water, and supplemented with 2 μg/ml DAPI and 0.2 μg/ml Nile red, which was then molded into a 125 μl GeneFrame (AbGene, Surrey, UK) to ensure even focus of the *z*-plane. Low OD_600_ samples were concentrated with a bench top centrifuge and re-suspended to an OD_600_ of ~0.5 in PBS buffer. 0.3 μl cells were spotted onto a slide and excess moisture was allowed to evaporate by resting the slide on a 37°C heating block before applying the coverslip. *L*. *lactis* strains were incubated with 0.05% TritonX-100 and 2 μg/ml DAPI for 5 min prior to immobilization on the microscope slides. TritonX-100 was added to facilitate the diffusion of DAPI into cells. Images were captured using a Nikon CoolSnap camera with a Zeiss Axiovert 200M epifluorescence microscope running MetaMorph software. Excitation/emission wavelengths and exposure times for fluorophores were as follows; 470/525 nm for 500 ms (GFP), 560/630 nm for 250 ms (Nile red), and 350/460 nm for 500 ms (DAPI).

Fully manual measurements of single-cell mean GFP intensities were carried out by using the ImageJ ‘ROI manager’, where straight line ROIs were hand-drawn along the central length axis of individual cells. For the cell diameter simulation, a through-focus stack with *z*-increments of 0.1 μm was acquired from 100 nm fluorescent beads to obtain realistic in- and out-of-focus light distributions.

### ObjectJ

Detailed information on the ImageJ plugin ObjectJ can be found at https://sils.fnwi.uva.nl/bcb/objectj. The use of ChainTracer and NucTracer is described in the results section and an extensive manual, including a description of embedded macros, is available as Supplemental Materials (S1 and S2 Files, respectively). A video demonstrating the use of ChainTracer is also available (S3 File). More information on both methods can be found at https://sils.fnwi.uva.nl/bcb/objectj/examples/ChainTracer/MD/chaintracer.html

## Results and Discussion

### ChainTracer project file

**Fig 1:**
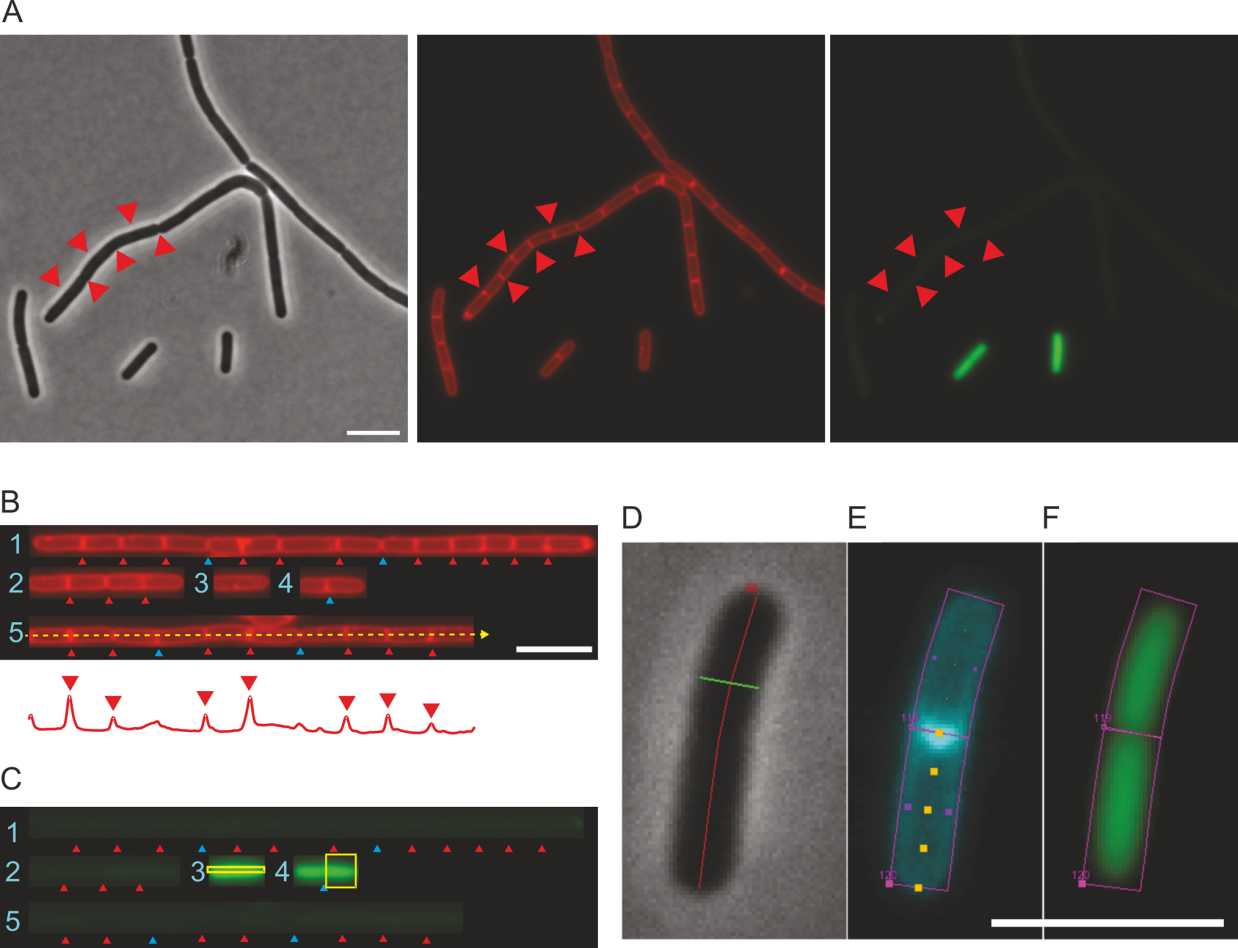
Microscopy images showing motile and non-motile *B*. *subtilis* cells during exponential growth in liquid medium. The *B*. *subtilis* strain (BSS339) contains the P_*hag*_*-gfp* reporter fusion. (A) Phase contrast, fluorescent membrane stain, and GFP images, respectively. Division septa, some indicated by red triangles, can be difficult to identify from phase contrast images. (B) ChainTracer screenshot showing straightened filaments. Filaments (*objects*) are numbered. An example of a line scan is shown as a dashed yellow arrow running through object #5, with resulting fluorescence intensity profile shown as a red graph underneath. Automatically detected peaks (septa) are indicated by red triangles over the intensity profile. (C) ChainTracer screenshot of the corresponding GFP channel. Automatically detected septa (*items*) are indicated by red triangles, manually added septa are indicated by blue triangles. The yellow boxes represent the two methods of fluorescence intensity data capture; a box encompassing an entire cell (cell #2 in filament #4) captures integrated GFP fluorescence, and a narrow box (cell #1 in filament #3) captures mean GFP measurement. (D-F) Summary of ObjectJ items making up an object in ChainTracer. (D) Cells shown in phase contrast, traced by a chain axis item (red), and bisected by a chain diameter (green) item. (E) Same cell as in D visualized in the membrane stain channel shows two cells, each bound by a cell box item (magenta), and an individual cell traced by a cell axis item (yellow dots). (F) Same cells as in D visualized in the GFP channel with cell box items (magenta). Scale bars are 5 μm.

*B*. *subtilis* cells can grow in chains and when viewed under phase contrast illumination they are difficult to outline by both automated and manual methods (Fig 1A, *B*. *subtilis* 168CA background). However, staining the cell membranes of these cells with a fluorescent dye will unambiguously reveal their outlines (Fig 1A). To our knowledge, no software exists that uses the cell membrane to identify bacterial cells in microscopy images. To this end, we developed ChainTracer using the ImageJ plugin ObjectJ [20]. ObjectJ extends the way user-defined regions of interest (ROIs) are handled. The plugin collects raw data, ROIs, results, and task-specific scripts into a self-contained ‘.ojj’ project file. The key advantage of ObjectJ is that users can easily navigate between analyzed data and the original images. Anomalies can be quickly detected, as ObjectJ allows for sorting by any measured property, so that the user can browse through cells with extreme values. Unwanted cell ROIs can then either be deleted or temporarily disqualified. The images are stored in a hyperstack format, which is a TIFF file containing multiple images arranged by channel and frame dimensions.

The use of ChainTracer is illustrated by measuring the expression of the flagellin promoter P*hag* in single cells [10]. ChainTracer is used with membrane-stained cells and carries out four semi-automated steps to obtain measurements of individual cells within a chain. In the first step, filamentous shapes (cell-chains) are identified from a phase contrast image, and are marked as ‘*chain objects*’. In the second step, the filaments are straightened to aid analysis (Fig 1B). In the third step, fluorescence intensity peaks along the chain axis in the membrane stain channel are used to automatically detect septa (Fig 1B). The septa are indicated by triangle markers placed along the cell-chain. In the fourth step, the chains are resolved into cells, which are marked as ‘*cell objects*’, which in turn have ‘*items*’ (e.g. cell axis and diameter markers) associated with them (Fig 1D–1F). Between each step, the user can manually check and modify objects and items via the graphical user interface. For example, septum positions can be added or deleted in cases where automatic detection has not worked (blue triangles in Fig 1B and 1C). Once individual cells have been identified, the amount of integrated fluorescence per cell in the green channel (GFP) is measured inside a box confined by two adjacent septa (Fig 1C, chain #4). Alternatively, the mean intensity along the cell axis can be measured (Fig 1C, chain #3). This may be preferable in cases where filaments form clusters, which can cause measurement boxes to overlap with a neighboring cell. All measured data are part of the results table contained in the ObjectJ project file, and can be exported to an external file. An example of such table is shown in the tutorial in the Supporting Information.

### Cell diameter underestimation

**Fig 2:**
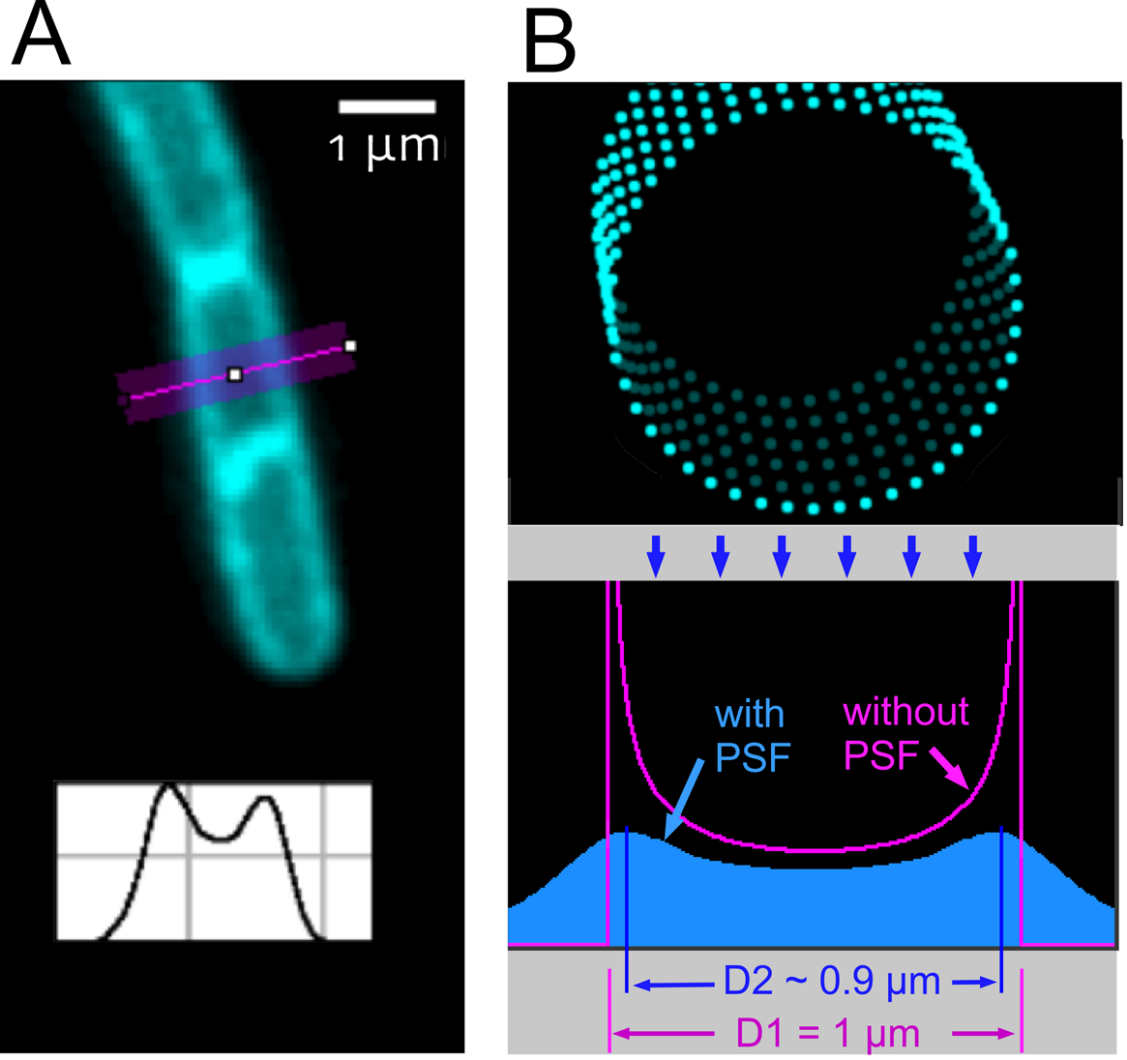
Simulation to quantify the difference between measured diameter and real diameter. (A) Membrane-stained cell observed with wide-field microscopy, with intensity profile perpendicular to the cell axis (inset). (B) Membrane is modeled as a three-dimensional tube composed of small fluorescent points (top) that are projected onto a two-dimensional plane. Two profiles were calculated, one without blurring effects (purple), and one with in- and out-of-focus blurring as defined by the 3D point-spread-function (PSF) of a real microscope (blue). The blurring effect decreases the distance between the maxima from D1 = 1μm to D2~0.9μm.

ChainTracer can measure lengths and diameters of individual cells. The cell diameter can be determined either from phase contrast images or by using the fluorescence of the stained membrane (Fig 2A). Although measurements based on fluorescence are usually more precise than those based on phase contrast, the outcome will give an underestimation of the cell diameter, as is illustrated in Fig 2. The image of a cell can be regarded as the projection of a three-dimensional object onto a two-dimensional surface. Since the projection is blurred by out-of-focus components and by the finite point-spread-function (PSF) of the microscope, the measured distance between the two peaks in Fig 2A is slightly smaller than the real diameter of the cell. This can be explained by the fact that smoothing a skewed peak moves its maximum position towards the less steep slope. Here, the unblurred intensity profile shows two peaks with less steep slopes towards the inside, and blurring decreases the distance between their maxima. The simulation first modelled a tube-shaped cell membrane consisting of fluorescent points. Then the light contribution of each point was derived from the microscope's 3D PSF for the corresponding in- or out-of-focus location, and was added to the projection area. As a consequence, the distance between the intensity maxima in the simulated profile decreased from 1.0 μm to ~0.9 μm. This systematic underestimation should be taken into account when measuring the cell diameter using membrane fluorescence.

### Single cell measurements with ChainTracer

**Fig 3:**
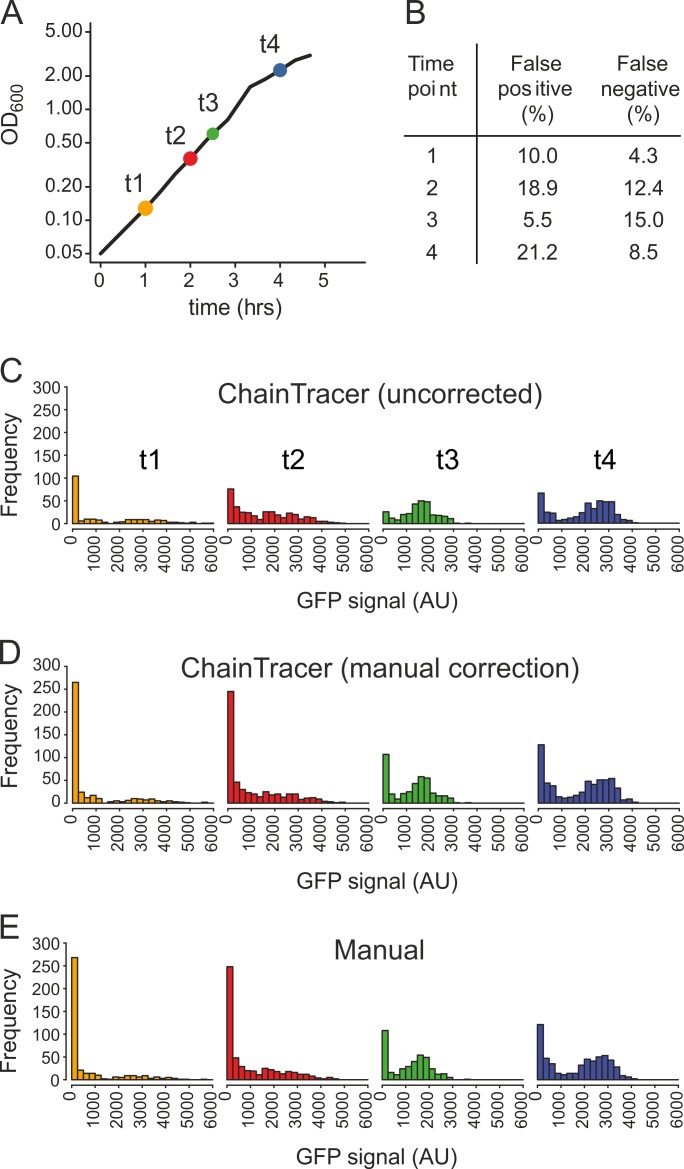
Bimodal expression of P_*hag*_*-gfp* during growth measured by ChainTracer. (A) Growth curve of BSS339 carrying the P_*hag*_*-gfp* reporter fusion grown in CH medium. Colored circles indicate time points where samples were collected for microscopy. Colors are re-used in subsequent Figs. (B) Table showing success rates for septum detection by ChainTracer for each time point. (C) Histograms of single-cell measurements obtained with ChainTracer showing GFP fluorescence intensities at time points 1 to 4 (n = 221, 369, 295, 427, respectively). (D) Data using ChainTracer with manual corrections between automated steps (n = 401, 544, 405, 585, respectively). (E) Manually acquired GFP fluorescence intensities at time points 1 to 4 (n = 397, 532, 386, 565, respectively). Y-axes show number of cells.

To test ChainTracer, we measured the induction of motility in a *B*. *subtilis* culture. The expression of motility genes is stimulated by positive feedback regulation leading to a bimodal response. During growth, an increasing number of cells become motile, and when the culture enters the stationary phase most *B*. *subtilis* cells are motile. Motility was monitored by using the flagellin promoter P_*hag*_ fused to *gfp* as reporter [10]. Samples of a growing *B*. *subtilis* culture were collected at 4 time points (Fig 3A), cells were stained with the fluorescent membrane dye Nile red, and imaged using fluorescence microscopy. During analysis by ChainTracer, roughly 1 in 10 septa were not detected (see e. g. Fig 1B) or were incorrectly specified (Fig 3B). It should be stressed that these values can be improved considerably by optimizing the conditions for membrane staining. For example, the use of freshly made fluorophore solutions and agarose slides reduce membrane staining artefacts. Fig 3C shows fluorescence intensity data resulting from running only the automated steps in ChainTracer. Data resulting after manual correction of misassigned septa are shown in Fig 3D. The higher cell numbers compared to Fig 3C are a consequence of septa that have escaped detection when ChainTracer is run without any manual input. The resulting histograms show clear bimodal distributions over time, with subpopulations exhibiting low and high GFP signal. This is in good agreement with previous reports [10]. To validate the accuracy of the ChainTracer data, a manual analysis of GFP intensities of individual cells was performed using ImageJ. As shown in Fig 3E, the distribution of values obtained from a fully manual analysis shows a good agreement with the ChainTracer data.

### Chain-length measurements

**Fig 4:**
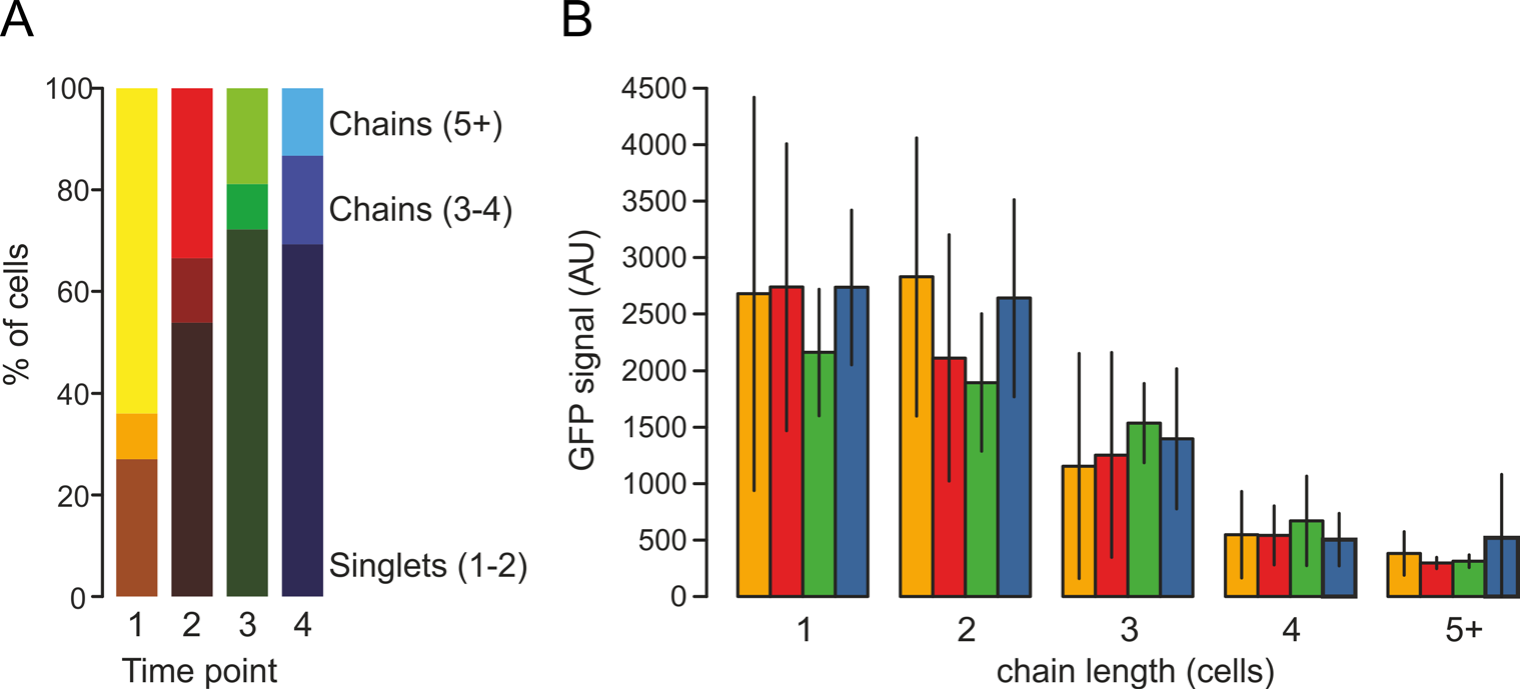
Cell-chain measurements using ChainTracer. (A) Stacked bar chart showing the distributions of chain lengths at the different time points of Fig 3D. Dark colors indicate singlets and doublets, intermediate colors indicate 3 or 4 cells, and light colors indicate chains of 5 or more cells, respectively. (B) P_*hag*_*-gfp* expression levels in relation to cell-chain length. Colors correspond to the different time points in Fig 3D. Error bars indicate standard deviations.

Development of motility is accompanied by the production of autolysins that help to release the cell from its non-motile siblings. ChainTracer records the hierarchy of *chain objects* and the number of *cell objects* they contain, which enables correlating single cell characteristics with cell-chain characteristics. We tested this with the data set used in Fig 3D. Cells were grouped into three classes; singlets and doublets, 3 or 4 cells joined together, and 5 or more cells joined together. Fig 4A shows how a culture shifts from predominantly longer cell-chains, to single or double cell-chain configurations. Fig 4B shows that the induction of flagellin production is strongly correlated with a reduction in chain length.

### Single cell measurements with NucTracer

**Fig 5:**
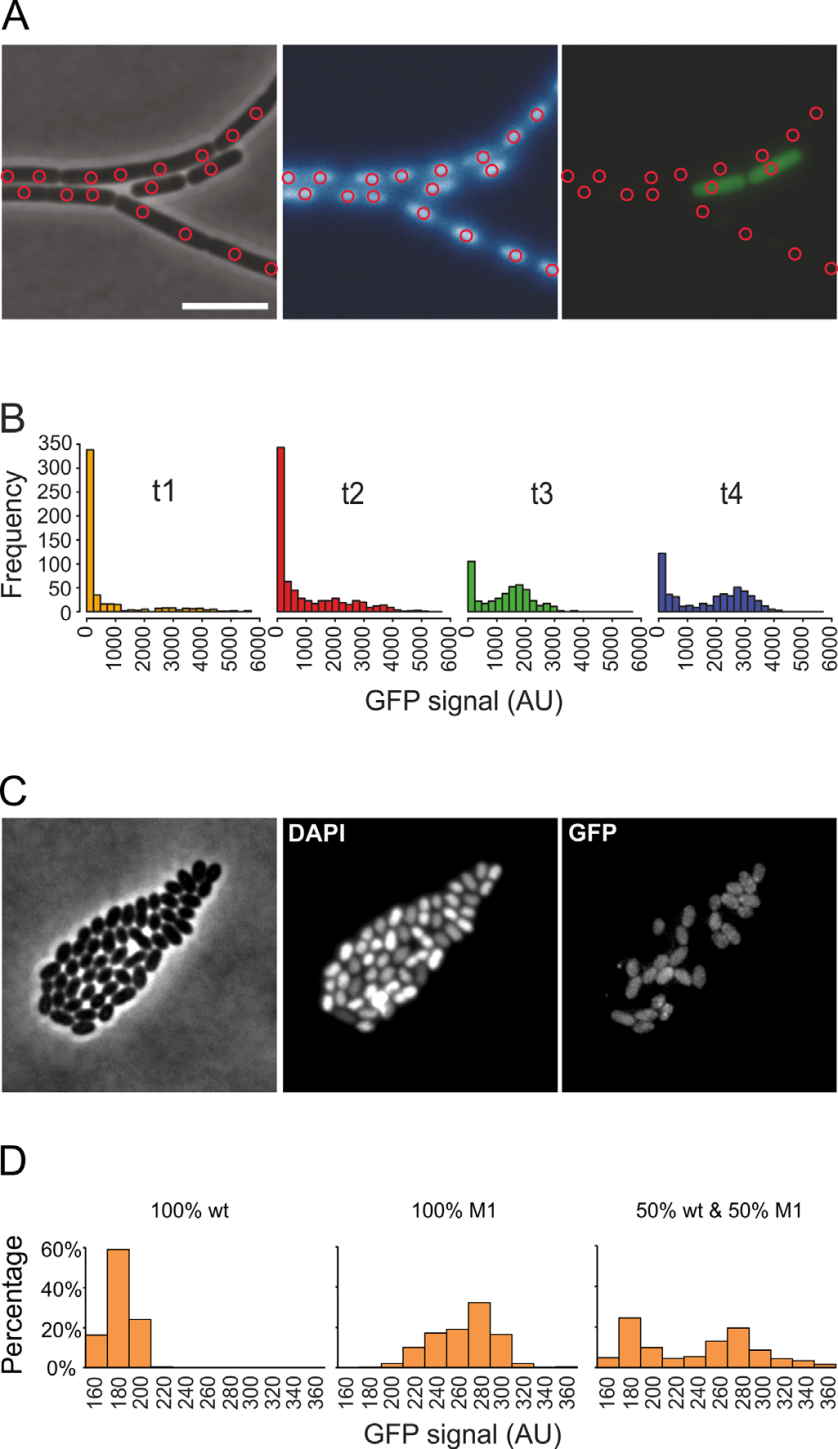
Single cell measurement using DAPI staining and NucTracer. The dataset of Fig 3 was used to identify cells based on DAPI stained nucleoids. (A) Phase contrast, DAPI and GFP channels, respectively, of the same microscopy image. Red circles indicate locations of intensity peaks in the DAPI channel that are used to collect GFP fluorescence intensity values. Scale bar is 5 μm. (B) Histograms of GFP fluorescence intensities collected using NucTracer (*n* = 496, 725, 454, 524, respectively). Colors correspond to the time points in Fig 3. (C) Phase contrast, DAPI and GFP image of a 1:1 mixture of wild type *L*. *lactis* cells and *L*. *lactis* cells expressing GFP (strain M1). (D) Histograms of GFP fluorescence intensity distributions obtained with NucTracer.

There are some drawbacks using membrane staining to identify cells. Firstly, many fluorescent membrane dyes such as FM4-64 and Nile red, have excitation and emission spectra that are incompatible with red fluorescence reporter proteins (e.g. mCherry). Secondly, performing relevant statistical testing often requires large cell numbers, and crowded microscopy images are preferred. However, the automated detection of cell-chains by ChainTracer requires that they should not make contact, which is difficult with high cell numbers. Therefore, we developed NucTracer to measure gene expression in single cells. This method uses the nucleoid stain as a proxy for single cells (Fig 5A). The advantages are that: (i) the commonly used nucleoid stain DAPI is compatible with both GFP and red fluorescent proteins, (ii) nucleoids of different cells are always physically separated, which allows for high cell densities, (iii) nucleoids are always located at the center of the cell, which is important for GFP intensity measurements, and (iv) nucleoid shape is less affected by cell shape. Of course, a cell often contains two nucleoids and therefore this method does not identify single cells in absolute terms. However, when a sufficiently large sample of cells is counted, this method will present an accurate picture of the cellular distribution in gene expression.

NucTracer was tested by analyzing the same microscopy images used in Fig 3. This was possible since the cells were stained both with Nile red and DAPI (see Materials and Methods for details). NucTracer detects local maxima of DAPI intensity, and uses a small circular sampling area with a diameter of 0.66 μm for each GFP intensity measurement (Fig 5A). Fig 5B shows the GFP intensity distribution over the nucleoid-determined ROIs at the 4 different time points without any manual correction. The distribution is clearly bimodal and is comparable to the completely manual measurements shown in Fig 3E. The number of ROIs is clearly higher than the number of cells (compare *n* in legends Fig 3 and Fig 5), but this difference does not influence the overall distribution of fluorescence intensities.

To show that NucTracer can be used with bacterial species of different shapes, the distribution of GFP expressing cells in the coccoid shaped bacterium *L*. *lactis* were measured. Wild type and GFP-expressing *L*. *lactis* cells were grown to log phase and mixed in equal numbers and stained with DAPI (Fig 5C). As shown in Fig 5D, analysis with NucTracer easily resolves the two subpopulations. Since we used a small amount of TritonX-100 (0.05%) to facilitate diffusion of DAPI over the cell membrane, the differences in DAPI signals are likely due to differences in the DNA content of cells.

### Comparison of both methods

**Fig 6:**
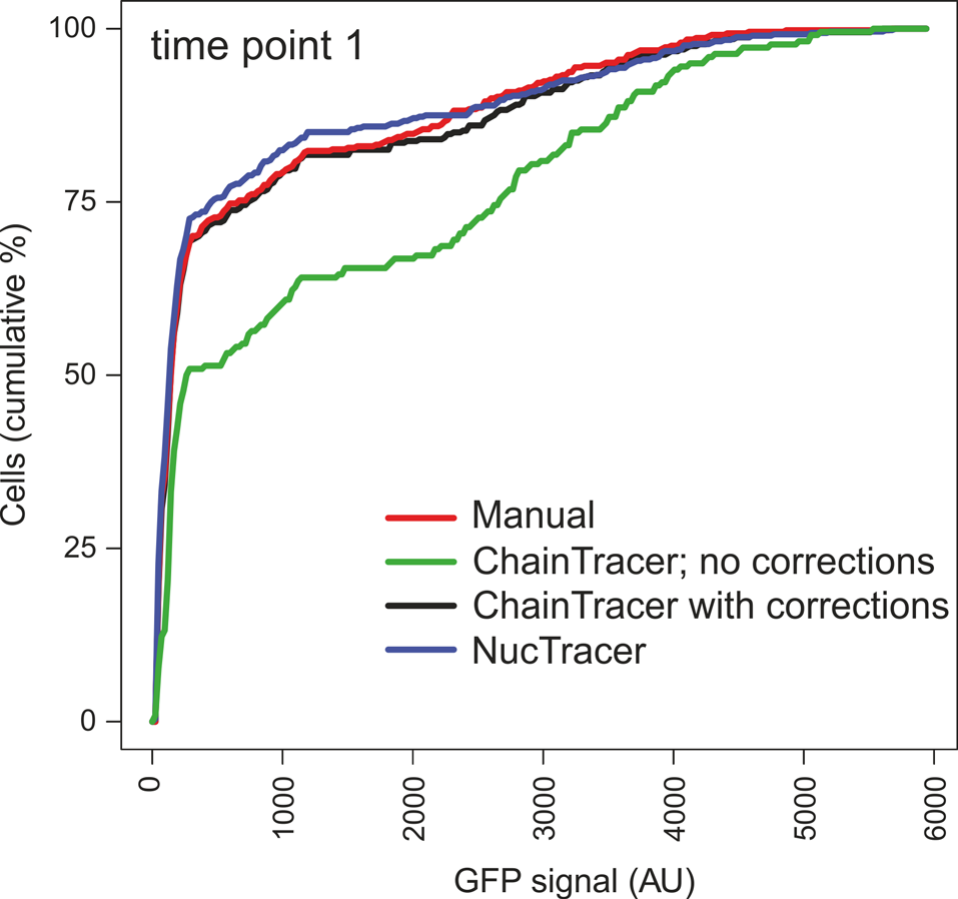
Comparison of NucTracer with ChainTracer. The cumulative frequency graphs show the distribution of fluorescence intensities at time point 1 (Figs 3C–3E & 5B) using ChainTracer (green), ChainTracer with manual correction (black), NucTracer without manual correction (blue), and manual measured fluorescence intensities using ImageJ (red). Cumulative frequency graphs were obtained by summing up the relative cell numbers (frequencies) for increasing fluorescence intensities.

To better compare the data of ChainTracer and NucTracer, the cumulative fraction of cells was plotted against increasing GFP fluorescence intensities for time point 1 of Fig 3 (see cumulative frequency graphs in Fig 6). The uncorrected ChainTracer data was also included (green line). Clearly, ChainTracer requires manual correction of septa identification to give accurate data, whereas the automated analysis by NucTracer gives reliable data that does not have to be checked manually. This shows another advantage of NucTracer. Of course, this is only true when microscopy images of sufficient quality are obtained.

### Conclusion

We have developed two methods that can be used to automatically measure gene expression in cells that form chains and that are difficult to distinguish by phase contrast. The advantage of the membrane staining method (ChainTracer) is that cell and chain dimensions can be determined. The advantage of the nucleoid staining method (NucTracer) is that it is compatible with both green as well as red fluorescent reporter proteins, that crowded images can be quickly analyzed, and that cell shape is less critical.

## Supporting Information

S1 File ChainTracer manual. (PDF)

S2 File NucTracer manual. (PDF)

S3 File ChainTracer video instruction. (MP4)
