# Supplementary material for: When Phase Contrast Fails: ChainTracer and NucTracer, Two ImageJ Methods for Semi-Automated Single Cell Analysis Using Membrane or DNA Staining
Source: PLoS One. 2016 Mar 23;11(3):e0151267. doi: 10.1371/journal.pone.0151267 (PMC4805268; doi:10.1371/journal.pone.0151267)
Supplement: S1 File — (PDF) [file pone.0151267.s001.pdf]

[All ObjectJ Examples](#)

ChainTracer

26-Oct-2015

[History](#)
[Download](#)

[Watch the Video](#) *(4:50 min, 11 MB, with sound)*

ChainTracer is a project that analyses multi-channel images of chain-forming bacteria.
It runs in combination with ImageJ and the ObjectJ plugin.
For cases where cells are too crowded, look for [NucTracer](#)

Norbert Vischer
Bacterial Cell Biology
University of Amsterdam

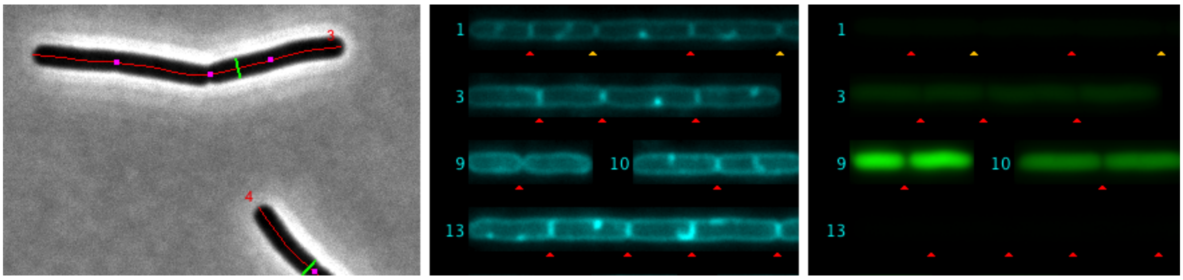

Features

- Marks axes and diameters of bacterial chains
- Resolves and marks individual cells
- Marks cell diameter from membrane fluorescence
- Measures GFP amount per cell in GFP channel
- Full back-and-forth navigaton

A. Installation of ImageJ and ObjectJ:

1. If not done yet, install ImageJ from: http://imagej.nih.gov/ij/
2. If the ObjectJ plugin is already visible under menu **Plugins**, jump to (4).
   Otherwise, you can install ObjectJ as follows:
   Download objectj_.jar from https://sils.fnwi.uva.nl/bcb/objectj/download/current/
   Make sure `objectj_.jar` was not renamed (because an older version already existed in the download folder).
3. Drag `objectj_.jar` onto ImageJ's main window (which contains the tools).
   Confirm when ImageJ asks to store this plugin in the plugins folder, and relaunch ImageJ.
4. You can choose **Plugins> ObjectJ** to make the **ObjectJ** menu visible (see fig 2),
   but the ObjectJ menu will appear anyway the first time you open an `.ojj` project file.

B. Run the demo:

1. [Download](#) and unpack the folder 'ChainTracer', which contains two hyperstacks. We will use it as our 'project folder'.
2. Download and unpack the project file 'ChainTracer-xx.ojj' and store it in your local project folder.
3. Your project folder will now look similar to this:

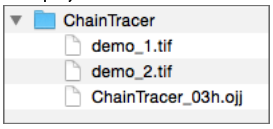

4. In order to open ChainTracer-xx.ojj, you can either drag its file icon from the Finder/Explorer into the main window with title "ImageJ", or you can use ImageJ's **Open** menu.
5. If the **ObjectJ** menu (between Analyze and Plugins, Fig 2) was not visible yet, it should appear now.
   Now the project window is shown, with the panel for linked images still being empty.
   In order to link the two files `demo_1.tif` and `demo_2.tif`, go to the Finder/Explorer and drag their icons into "Linked Images" panel (Fig 2).
   Alternatively, you can link images via menu **ObjectJ>Linked Images**.
   The two green bullets confirm that the image files are in the same folder as the project.
   The hyperstacks used here contain several channels and frames. Channel 1 (gray) is used for phase contrast, channel 2 (cyan) for membrane

staining, and channel 3 (green) for GFP. Now you can start with the analysis, using the embedded macro commands under ObjectJ menu.

Detecting Chains

6. Choose **ObjectJ> Mark Filaments.** This is the first embedded macro.
   It measures and marks all phase contrast images contained in the two hyperstacks, and indicates where criteria are not met in case of rejection (Fig 3)

7. Choose **ObjectJ> Make Straight Cells**
   All marked cell chains are arranged in a single hyperstack called "StraightCells.tif" and saved in the project folder. Chain number labels are shown as non-destrucitive overlay, that is visible in all three channels.

Detecting Septa

8. Choose **ObjectJ> Find All Septa**
   In "Straight Cells", Channel 2 is evaluated to detect septa. These are marked as non-destructive red triangles.

Modifying Septa

9. Manually add or delete septum markers
   You can add more septa manually by locating the cursor at the desired position in "Straight Cells" and then press key "A". This is the shortcut for macro **ObjectJ> Add Septum**. A manually added septum is marked with a yellow triangle.
   Similarly, you can delete a septum with the key "D" (macro **ObjectJ> Delete Septum [D]**).

10. You can dispay the intensity profile that is used to distignuish septa by placing the cursor upon a cell and press key "F" (macro **Show Profile [F]**).

Resolving Cells

11. Choose **ObjectJ> Resolve Cells from Chains** to mark individual cells. A cell is characterized by a cell axis (segmented line, only vertices are visible as dots), and a cell box that uses the diameter of the cell chain.

Measuring Cells

12. Choose **ObjectJ> Calc Diameters via Membrane** to set markers that indicate the cell diameter as derived from the membrane stain. For marking, a line object is used, but only the end points are visible as dark purple dots.

13. Choose **ObjectJ> Calc GFP Segments** to output the desired fluorescence values per cell. For this measurement, the cell box is applied to the GFP channel to calculate integrated fluorescence.

14. Choose **ObjectJ> Save Project** to save the project.

Navigating

15. You can use the commands under **ObjectJ> Navigate>** to travel through (sorted) objects in an ordered way, or cycle through channels with a single key stroke.

Deleting Chain Objects

16. Chain markers do not disturb the statistics for individual cells. Still there may be reasons to delete chain markers to simplify the ObjectJ results table. This can be done by disqualifying (e.g. "qualify if CellAxis exists" will disqualify non-cells) them and then delete all disqualified objects. You cannot edit any septum position after that.

C. Screenshots:

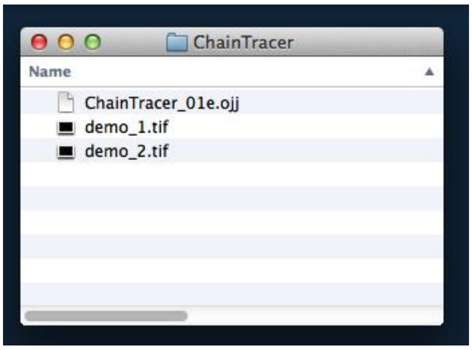

Fig 1: Project file (ending with .ojj) and images need to be in same folder

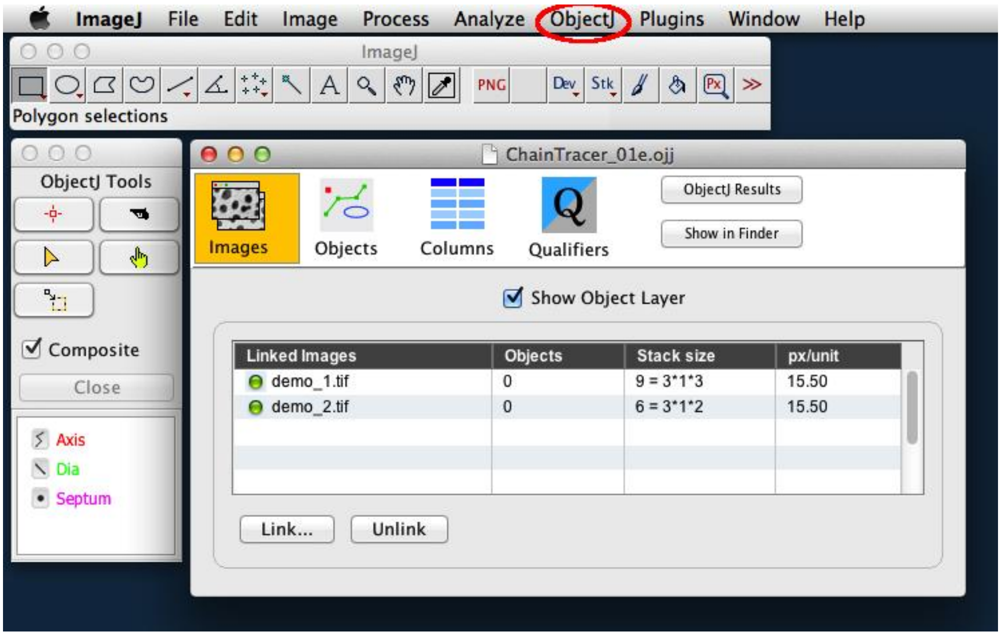

Fig 2: *Left:* ObjectJ Tools indicates item types Axis, Dia, and Septum that are used for marking.
Right: 'Linked Images' panel of project window shows that two stacks (demo_1.tif and demo_2.tif) are linked. Green bullets indicate that the files are in the correct folder.

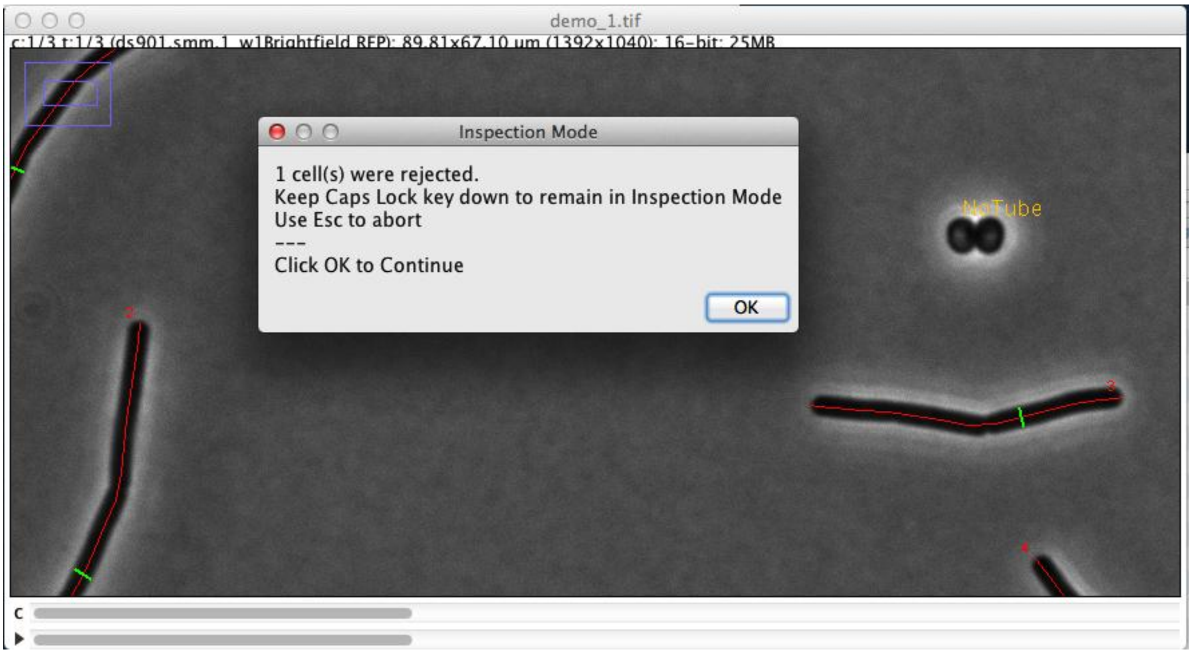

Fig 3: Automatic marking of cell chain axis (red) and diameter (green). Rejected cells are temporarily labeled in yellow.

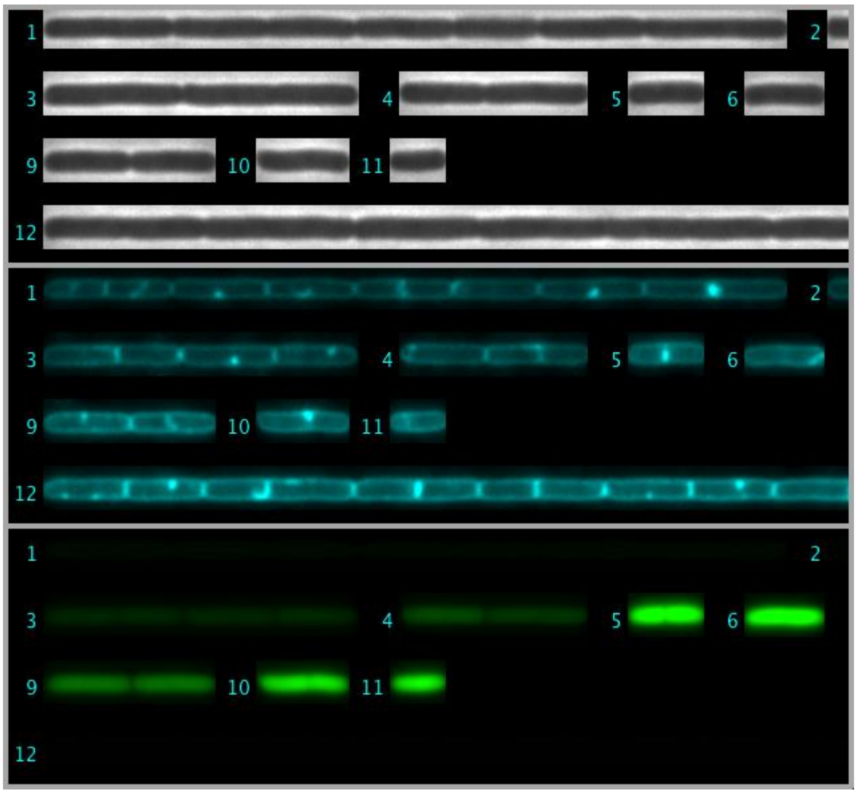

Fig 4: Straightened cell chains are arranged as 3-channel hyperstack. This file is saved in the project folder.

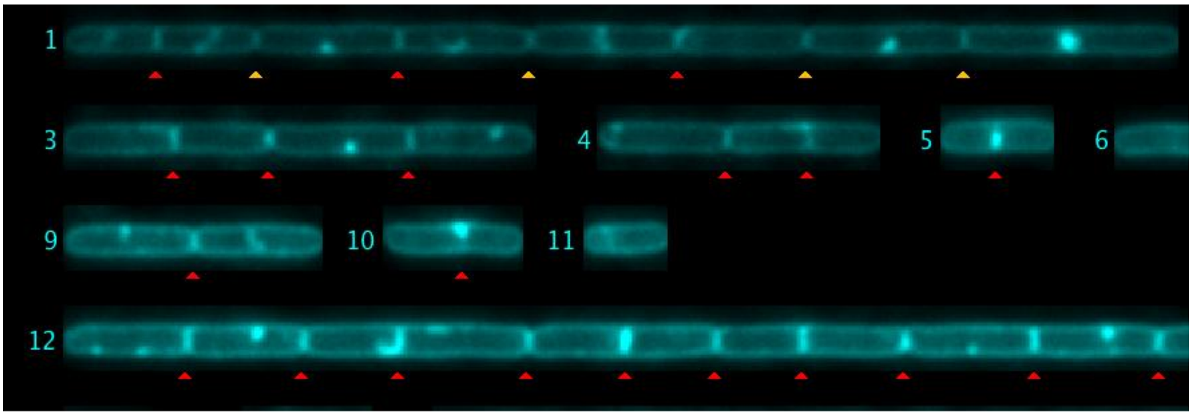

Fig 5: Septa are marked automatically with red triangles. In chain #1, four yellow triangles were added manually. Also, some red triangles (false positives) were removed manually.

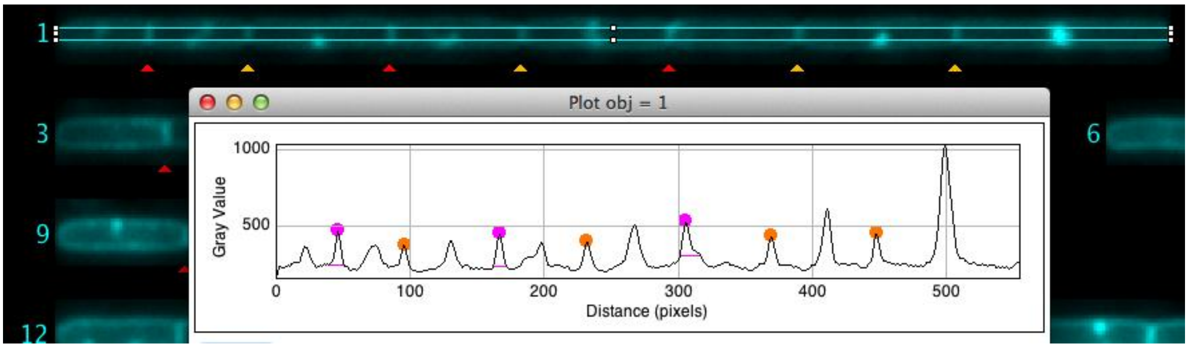

Fig 6: Locating the cursor on cell chain #1 and pressing key **"F"** shows its intensity profile with marked peaks. Small horizontal magenta lines indicate the noise level used to separate peaks. Some peaks on the right were manually unmarked.

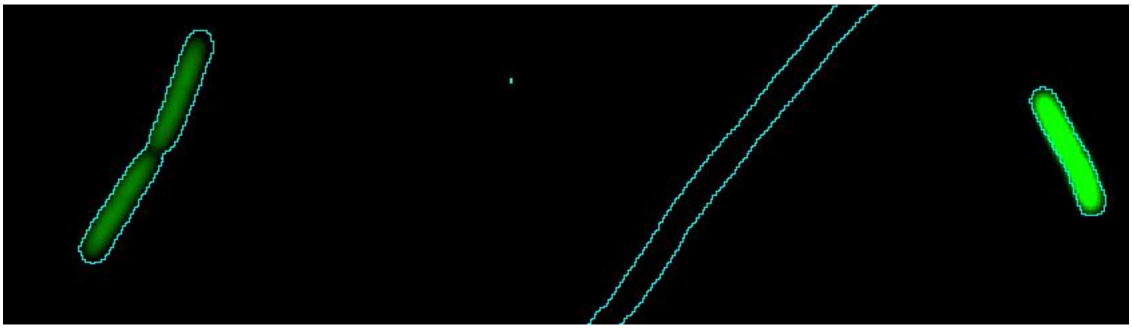

Fig 7: Cell outlines as derived from the phase contrast image can be shown in other channels via command **ObjectJ Show / Hide Outlines**.

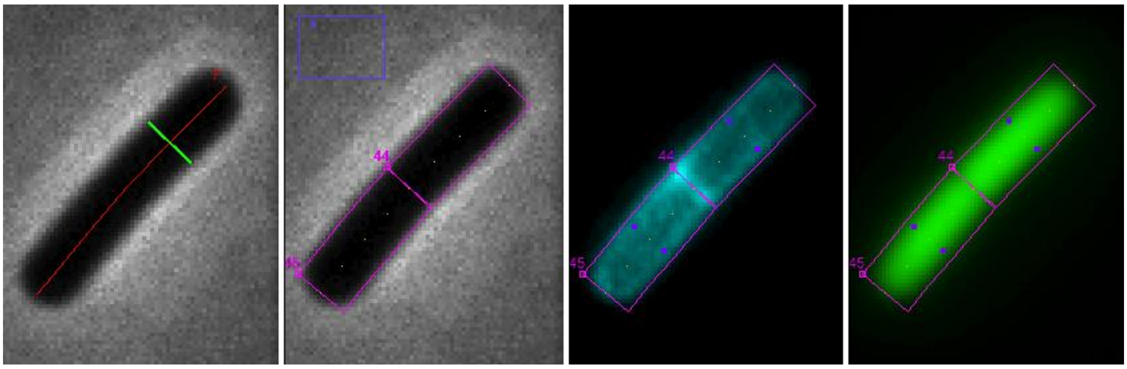

Fig 8: **ObjectJ> Marking Chains and Cells** Chain is marked with red axis line and green diameter. By choosing 'Resolve Chains to Cells", additional cell objects are marked(magenta: cell box, yellow dots: cell axis, purple dots: diameter of membrane cylinder)

ObjectJ results

Linked results | Unlinked results

[Stat]	Axis	Dia	CellAxis	DiaP	CellDiaM	GFPfluor	GFPMean	GFPMax
Count	27	27	104	104	103	104	104	104
Mean	11.77	0.89	3.05	0.88	0.72	1.82	1392.40	3405.01
StDev	14.28	0.04	1.03	0.03	0.05	2.13	1623.65	3967.39
78			2.96	0.85	0.65	4.83	3184.34	7700.00
79			2.81	0.85	0.65	4.65	3200.94	7835.00
80			2.12	0.89	0.65	4.63	4247.85	9982.00
81			1.38	0.89	0.66	2.92	4016.40	9895.00
82	3.89	0.96						
83	4.18	0.89						
84	36.85	0.85						
85	6.76	0.86						
86	3.67	0.94						
87	5.12	0.96						
88	6.30	0.89						
89	4.41	0.89						
90	6.48	0.90						
91	4.61	0.90						
92	4.95	0.88						
93	6.82	0.86						
94	3.68	0.90						
95			3.88	0.96	0.76	7.59	3768.04	9805.00
96			2.32	0.89	0.71	1.77	1488.09	3786.00
97			1.86	0.89	0.66	1.46	1522.11	3754.00
98			3.73	0.85	0.71	0.79	413.32	1024.00
99			3.68	0.85	0.71	0.82	436.96	1032.00

Fig 9 : **ObjectJ> Show ObjectJ Results** shows linked results. Objects of type 'Chain' and type 'Cell' can co-exist in one table, as they occupy different columns. For example, object #81 is a cell and object #82 is a chain.

D. Menu Commands:

This project has embedded macros (macro text is editable via ObjectJ> Show Embedded Macros), which appear indented in the ObjectJ menu:

Mark Filaments

Marks chain axis and mean diameter of the cell chain. Marking takes place in phase contrast channel of all linked images. Reasons for rejected cells are temporarily shown as orange text overlay. The ObjectJ results table is automatically populated with corresponding values (e.g. Axis = 20 um, Dia = 0.9 um).

Make Straight Cells

All marked chains are straightened and arranged in a single hyperstack called "StraightCells.tif", which is saved in the project folder. Cell number labels are shown as non-destrucitive overlay, that is visible in all three channels.

Show Straight Cells
Opens StraightCells.tif from the project folder, or brings it to the front if it was open.

Find All Septa
Septa are marked automatically in "Straight Cells" with red triangles by evaluating profiles in channel 2 (Membrane stain). Positions of septa are stored in the project file.

Add Septum [A]
This macro can only be used via the shortcut key "A". It adds a yellow triangle at the cursor position.

Delete Septum [D]
This macro can only be used via the shortcut key "D". It deletes the triangle under the cursor position.

Delete Cells or Chains..
Deleting cells may be useful when you want to go back and edit septum positions, and then resolve cells from chains again. Deleting chains may be useful to simplify the ObjectJ results table, when chain information is not needed anymore.

Rebuild Overlay
Reads the septa positions from the project file and re-paints red or yellow triangles into "Straight Cells".

Select Back-Forth [S]
This macro can only be used via the shortcut key "S". If StraightCells is in front, the evaluation rectangle under the cursor is displayed, and the correponding object is highlighted in both the original stack and in the ObjectJ results table.
Similarly, if a hyperstack is in front with a selected cell or chain, its straightened version is brought to front.

Show Profile [F]
This macro can only be used via the shortcut key "F". It draws an intensity profile along the cell chain in the membrane channel. The peaks in the profile are highlighted in red (automatic) or yellow (manual).

Hide/Show Outlines [[F6]
Cell outlines as derived from the phase contrast image are be shown in the current channel.

Resolve Cells from Chains
Uses detected septa to create new cell objects with their own number labels. A cell object consist of a cell axis (segmented line), and a cell box.

Calc Diameters via Membrane
Loops through all cell objects and determines the diameter of the cylindrical part of the cell membrane. This is done by finding the maxima of membrane fluorescence using several perpenidcular profiles.

Calc GFP Segments
Uses the cell boxes to determine integrated fluorescence in the GFP channel.

Subtract Fluor. Backgrounds ..
From all linked fluorescent channels, backgrounds are calculated (as modal value of central 80% of width and height). With key Caps Lock down, the observer will see those pixels in red which would become zero after background subtraction. Before performing any subtraction, a table of calculated backgrouds is shown, with the option to edit the table or abort the process. The table is also copied to the clipboard, and if the user chooses File>New>System Clipboard, there are more options to edit than only to delete rows. However, the table columns may not be properly aligned.

E. ObjectJ Result Columns

Column	*Explanation*
**ChainAxis**	Axis length of cell chain
**ChainDia**	Diameter of cell chain (calculated from area and axis length)
**CellAxis**	Length of individual cell
**DiaP**	Diameter of the cells parent chain
**CellDiaM**	Diameter of the cells membrane cylinder
**GFPFluor**	Integrated GFP fluorescence inside cell box (x0.001)
**GFPMax**	Max GFP fluorescence inside cell box
**GFPMean**	Mean GFP fluorescence inside cell box
**GFPMid**	Mean GFP fluorescence inside along the cell axis
**ParentID**	Negative ids for chains, positive ids identify a cell's parent

ChainTracer: Embedded Macro Commands

macro "Mark Filaments"
Marks filaments in those images that are linked but not marked yet. We first calculate a threshold, then analyze particles and store their rois in the roi manager. Subsequently, each roi is isolated as mask (everything else black) and traced by a perpendicular slit window. Markers are set along the axis, diameter (calculated from area and length) is marked

macro "Make StraightCells"
Arranges all cells straightened in a single hyperstack.
A temporary monitor window visualises this with live scrolling

macro "Show Straight Cells"
shows Image "StraightCells", or reads it from disk

macro "Find All Septa"
Creates a profile along the axis of each chain and marks the maxima as septa. In fact, two profiles are created on either side of the axis in order to measure symmetry and reject single-sided blobs.

macro "Add Septum [A]"
Manually adds septum triangle at cursor position

macro "Delete Septum [D]"
Manually deletes septum triangle from cursor position

macro "Delete Cells or Chains..."
Removes only cell objects or only chain objects

macro "Rebuild Overlay"
Rebuilds Ooerlay on StraightCells (chain number and triangles indicating septa).
Chain numbers are rebuilt using columns BarX, BarY,
Septa triangles are rebuilt via columns BarX, BarY, SeptaPos

macro "Select back-forth [S]"
If you are in StraightCells, select and show chain in linked stack.
Otherwise, jump to StraightCells and expose selected chain.

macro "Show Profile [F]"
If you are in StraightCells, from the chain under the cursor position
an intensity profile with detected maxima is shown.

macro "<Navigate>Zoom to previous Object   [F1]"
shows previous object zoomed-in (respects qualifying and sorting)

macro "<Navigate>Zoom to next Object   [F2]"
shows next object zoomed-in (respects qualifying and sorting)

macro "<Navigate>Zoom to current Object   [F3]"
shows currrently selected object zoomed-in

macro "<Navigate>Zoom to Object#...   [F4]"
shows object # xx zoomed-in

macro "<Navigate>Next Channel   [F5]"
All markers reside on channel 1, but they can be made visible by
increasing the visibility depth. This is done here, and the active channel

is incremented, or jumps back to #1.

macro "<Navigate>Show Composite Ch1+Ch2 [Z]"
Shortcut to show only channel #1 and #2 in composite mode,
with channel #1 in blue color (i.e. dark end less disturbing)

macro "Show/ Hide Outlines [F6]"
Reads the threshold of the current image from ObjectJ Results
Applies the threshold and creates all cell contours.
(Toggling on/off)

macro "Resolve Cells from Chains"
The segmented lines that mark the chain axes are split into smaller parts (cells)
by reading the septa positions from the ObjectJ results.
A cell axis (part of the chain axis) is then described by a line consisting of
four segments. The parent chain of a cell is recorded as the parentID, which is the
absolute value of the (negative) ID of the parent chain.

macro "Calc Diameters via Membrane"
Creates fluorescence profile perpendicular to the
cell axis, and sets point markers at the maxima.
The distance between the markers corresponds to the diameter
of the tubular membrane. This value is underestimated and should be corrected.

macro "Calc GFP Segments"
calculates integrated fluorescence in rectangular rois (using column "SeptaPos")
in GFP channel of straight cells.

macro "Subtract Fluor. Backgrounds ..."
Opens each linked stack. Channel #1 is assumed to be phase contrast.
The other channels are assumed to be fluorescent images,
from which the individual background (=modal value) will be calculated.
Only central 80% of width and of height are used for this calculation.
Before actually subtracting backgrounds and saving, user can delete
rows from the table, or abort macro via 'Esc' key.
