# Supplementary material for: When Phase Contrast Fails: ChainTracer and NucTracer, Two ImageJ Methods for Semi-Automated Single Cell Analysis Using Membrane or DNA Staining
Source: PLoS One. 2016 Mar 23;11(3):e0151267. doi: 10.1371/journal.pone.0151267 (PMC4805268; doi:10.1371/journal.pone.0151267)
Supplement: S2 File — (PDF) [file pone.0151267.s002.pdf]

[All ObjectJ Examples](#)

NucTracer

26-Oct-2015

[Download](#)

NucTracer is a simple project that analyses fluorescent peaks in multi-channel images of bacteria.
It can be used where cells are too crowded to be detected in phase contrast.
Rather than finding cell contours, DAPI stain is used to localize the centers of individual cells.
- See also [ChainTracer](#)

Norbert Vischer
Bacterial Cell Biology
University of Amsterdam

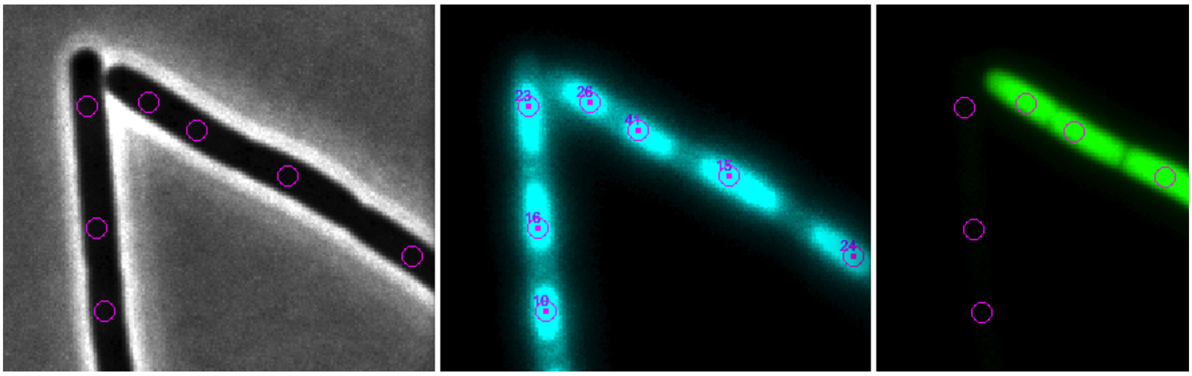

Features
- uses DAPI stained channel to mark fluorescent maxima
- samples peak fluorescence in all fluorescent channels

A. Installation of ImageJ and ObjectJ:

1. If not done yet, install ImageJ from: http://imagej.nih.gov/ij/
2. If the ObjectJ plugin is already visible under menu **Plugins**, jump to (4).
   Otherwise, you can install ObjectJ as follows:
   Download objectj_.jar from https://sils.fnwi.uva.nl/bcb/objectj/download/current/
   Make sure `objectj_.jar` was not renamed (because an older version already existed in the download folder).
3. Drag `objectj_.jar` onto ImageJ's main window (which contains the tools).
   Confirm when ImageJ asks to store this plugin in the plugins folder, and relaunch ImageJ.
4. You can choose **Plugins> ObjectJ** to make the **ObjectJ** menu visible,
   but the ObjectJ menu will appear anyway the first time you open an `.ojj` project file.

B. Run the demo:

1. [Download](#) and unpack the folder 'NucTracerDemo', which contains two hyperstacks. We will use it as our 'project folder'.
2. Download and unpack the project file 'NucTracer-xx.ojj' and store it in your local project folder.
3. Your project folder will then look similar to this:

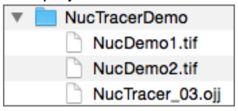

4. In order to open NucTracer-xx.ojj, you can either drag its file icon from the Finder/Explorer into the main window with title "ImageJ", or you can use ImageJ's **Open** menu.
5. If the **ObjectJ** menu (between Analyze and Plugins) was not visible yet, it should appear now.
   Now the project window is shown, with the panel for linked images still being empty.
   In order to link the two files `NucDemo_1.tif` and `NucDemo_2.tif`, go to the Finder/Explorer and drag their icons into "Linked Images" panel.
   Alternatively, you can link images via menu **ObjectJ>Linked Images**.
   The two green bullets confirm that the image files are in the same folder as the project.
   The hyperstacks used here contain several channels and frames. Channel 1 (gray) is used for phase contrast, channel 2 (cyan) for membrane staining, and channel 3 (green) for GFP. Now you can start with the analysis, using the embedded macro commands under ObjectJ menu.

Detecting Fluorescent Peaks

6. Choose **ObjectJ>Mark Cells in DAPI [1]** This is the first embedded macro.
   It measures and marks all DAPI images contained in the two hyperstacks by setting markers onto fluorescent peaks.

7. Choose **ObjectJ> Measure GFP and DAPI [2]**
   Using the markers from the previous step, teh mean intensitisz inside circles are recorded in GFP and DAPI channels

8. Choose **ObjectJ> Show/Hide Circles [3]**
   Visualises the circular selections which are used to calculate the mean fluorescence per cell.

9. Choose **ObjectJ> Show ObjectJ Results**
   Shows linked results. For statistics, click on "Stat". For histograms, sorting or other column operations, right-click a column title.

C. ObjectJ Result Columns

Column	***Explanation***
**FluorGFP**	Mean GFP fluorescence inside each circle
**FluorDAPI**	Mean DAPI fluorescence inside each circle

NucTracer: Embedded Macro Commands

macro "Mark Cells in DAPI [1]"
Works with 3-channel multi-frame hyperstacks
Marks maxima in channel 3 and then draws little circles
to calculate their means in channels 2 and 3.
Ch3 is filtered with Gauss (sigma = 2 px; only during maximum finder)
Auto-threshold is used to calculate noise tolerance for maximum finder:
tolerance = 0.1 * thresholdRange

macro "Measure GFP and DAPI [2]"
Measures mean intensity inside small circle in channel 2 and 3
It is assumed that the background is already subtracted

macro "Show/Hide Circles [3]"
Draws or hides small magenta circles that indicate the evaluation area per cell.
These circles are visible independent of current channel.
If circles of previous image or frame are shown, hide and show again.
